# Hemodynamic Response of the Supplementary Motor Area during Locomotor Tasks with Upright versus Horizontal Postures in Humans

**DOI:** 10.1155/2016/6168245

**Published:** 2016-06-19

**Authors:** Arito Yozu, Shigeru Obayashi, Katsumi Nakajima, Yukihiro Hara

**Affiliations:** ^1^Department of Rehabilitation Medicine, Graduate School of Medicine, Nippon Medical School, 1-1-5 Sendagi, Bunkyo-ku, Tokyo 113-8602, Japan; ^2^Department of Rehabilitation Medicine, Nippon Medical School Chiba-Hokusoh Hospital, 1715 Kamakari, Inzai, Chiba 270-1694, Japan; ^3^Department of Rehabilitation Medicine, The University of Tokyo Hospital, 7-3-1 Hongo, Bunkyo-ku, Tokyo 113-8655, Japan; ^4^Department of Physiology, Faculty of Medicine, Kindai University, 377-2 Ohno-Higashi, Osakasayama, Osaka 589-8511, Japan

## Abstract

To understand cortical mechanisms related to truncal posture control during human locomotion, we investigated hemodynamic responses in the supplementary motor area (SMA) with quadrupedal and bipedal gaits using functional near-infrared spectroscopy in 10 healthy adults. The subjects performed three locomotor tasks where the degree of postural instability varied biomechanically, namely, hand-knee quadrupedal crawling (HKQuad task), upright quadrupedalism using bilateral Lofstrand crutches (UpQuad task), and typical upright bipedalism (UpBi task), on a treadmill. We measured the concentration of oxygenated hemoglobin (oxy-Hb) during the tasks. The oxy-Hb significantly decreased in the SMA during the HKQuad task, whereas it increased during the UpQuad task. No significant responses were observed during the UpBi task. Based on the degree of oxy-Hb responses, we ranked these locomotor tasks as UpQuad > UpBi > HKQuad. The order of the different tasks did not correspond with postural instability of the tasks. However, qualitative inspection of oxy-Hb time courses showed that oxy-Hb waveform patterns differed between upright posture tasks (peak-plateau-trough pattern for the UpQuad and UpBi tasks) and horizontal posture task (downhill pattern for the HKQuad task). Thus, the SMA may contribute to the control of truncal posture accompanying locomotor movements in humans.

## 1. Introduction

Upright bipedalism is one of the distinctive features that differentiates humans from quadrupedal mammals. Recent advances in brain imaging techniques facilitate the investigation of brain activity levels during movement by unrestrained human subjects. Imaging studies using single photon emission computed tomography [1] and functional near-infrared spectroscopy (fNIRS) [2] have demonstrated that, in the frontal lobe, the primary sensorimotor area and the supplementary motor area (SMA) were coactivated during walking in healthy human subjects.

For successful execution of bipedal gait, the central nervous system (CNS) integrates neural substrates involved in the control of upright posture and stepping movements. Concerning cortical mechanisms for the control of locomotion, neurophysiological studies in cats and humans have demonstrated that the (primary) motor cortex modulates ongoing activities in the spinal circuitries via the corticospinal or the pyramidal tract [3, 4]. However, studies on the role of the SMA in locomotor control are fairly limited. Clinical observations have shown that patients with focal lesions involving the SMA exhibited mixed signs including disequilibrium and gait abnormalities [5, 6]. Gurfinkel' and Él'ner [7] and Viallet et al. [8] studied patients with brain lesions and hypothesized that the secondary motor area, particularly the SMA, may participate in postural adjustments associated with voluntary movements. However, experimental studies to test this hypothesis are greatly lacking. Thus, our understanding of the functional significance of the SMA in the control of bipedal gait in humans remains unclear.

Human upright bipedalism is generally accepted to have evolved from ancestral quadrupedalism [9]. In addition, humans commonly exhibit quadrupedalism during infancy, that is, crawling, which then develops into upright bipedalism. Experimental evaluations of the functional significance of the SMA in postural control during locomotion could include comparative studies of mammalian quadrupedalism and human upright bipedalism or among humans at different developmental stages, such as crawling in infants versus upright bipedalism in adults. However, these intergroup comparisons are applied onto the different CNS and musculoskeletal system across the groups; thus, they hardly extract the main posture-specific differences in different locomotor modes. By contrast, intragroup comparisons of quadrupedal and bipedal gaits are expected to be much more effective because the CNS and musculoskeletal system in a single subject can be studied in two modes of locomotion. However, there are currently no previous neuroimaging studies comparing bipedalism and quadrupedalism performed in the same subjects or even those only on quadrupedalism in humans.

The aim of the present study was to investigate the functional significance of the SMA in the control of locomotor behavior using fNIRS by focusing on posture. Quadrupedal stance is considered more stable than upright stance, because the center of gravity (COG) is closer to the ground and the base of support bounded by the hands and feet is larger than that by the left and right feet only. Based on these biomechanical perspectives, we hypothesized that the activation level of the SMA in human adults would correlate with degree of postural instability that accompanies locomotor movements. To test this hypothesis at a coarse-grain level, we asked each subject to perform three locomotor tasks, namely, hand-knee quadrupedal crawling (HKQuad task), upright quadrupedalism using bilateral Lofstrand crutches (UpQuad task), and typical upright bipedalism (UpBi task) on a treadmill, where the degree of postural instability varied biomechanically, and we measured hemodynamic responses in the SMA.

## 2. Materials and Methods

### 2.1. Participants

Ten healthy human adults (five males and five females aged 32.0 ± 7.7 (mean ± SD) years, range, 23–45 years) participated in this study. All participants provided their written informed consent prior to the study. All the procedures were conducted in accordance with the Declaration of Helsinki, and they were approved by the Ethics Committee of Chiba-Hokusoh Hospital, Nippon Medical School.

### 2.2. fNIRS

We used an fNIRS system (ETG-4000 Optical Topography System; Hitachi Medical Co., Tokyo, Japan) to measure the SMA activity, while participants performed locomotor tasks on a treadmill (Fitcrew GMJP-T1-65-12130001; Greenmaster Japan Co. Ltd., Tokyo, Japan). The details of the fNIRS system were described in our previous studies [10, 11]. In brief, the system emitted near-infrared light (695 and 830 nm) and detected the transmitted light to measure relative changes in oxygenated hemoglobin (oxy-Hb) and deoxygenated hemoglobin (deoxy-Hb) concentrations. The oxy-Hb value was measured every 0.1 s (i.e., sampling rate of 10 Hz).

**Figure 1:**
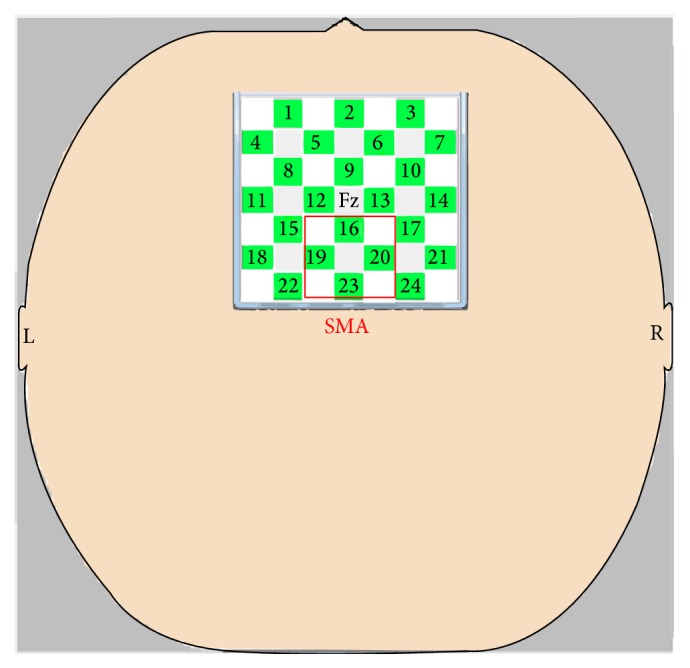
Near-infrared spectroscopy probe settings. The supplementary motor area (SMA) was covered by channels 16, 19, 20, and 23 within the red square.

Eight emission probes and eight detection probes (yielding 24 channels) were positioned with centering on Fz according to the international 10/20 system for electroencephalogram electrode placement (Figure 1). The interprobe distance was set at 3.0 cm. As reported by Okamoto et al. [12] and in our previous study [11], the SMA was covered by channels 16, 19, 20, and 23.

### 2.3. Tasks for fNIRS

**Figure 2:**
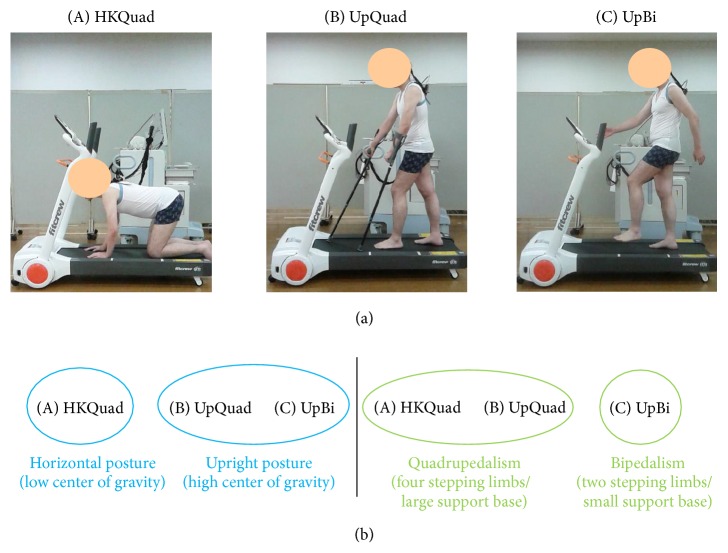
Experimental setup. (a) Images of three locomotor tasks during hand-knee quadrupedal crawling (HKQuad) (A), upright quadrupedalism (UpQuad) using bilateral Lofstrand crutches (B), and usual upright bipedalism (UpBi) (C). (b) The conceptual framework for the task design. Differences in the height of the center of gravity distinguished between HKQuad and the other two tasks (UpQuad and UpBi) (left). The difference in the number of stepping limbs that encompassed the support base distinguished between UpBi and the other two tasks (HKQuad and UpQuad) (right).

All participants were required to perform three locomotor tasks on the treadmill: hand-knee quadrupedal crawling (HKQuad task), upright quadrupedalism using bilateral Lofstrand crutches (UpQuad task), and typical upright bipedalism (UpBi task) (Figure 2(a)). From biomechanical perspectives, the stability of the stance is proportional to the inverse of the height of the COG, area of the base of support, and weight of the body [13]. Thus, we designed the three tasks so that the combinations of the area of the support base (large or small) and the height of the COG above it (high or low) differed.

In the HKQuad task (Figure 2(a), (A)), participants were required to crawl quadrupedally with a dorsal-side-up posture. In this task, the trunk was maintained nearly horizontal (low COG) and supported by all four limbs (large support base). It has been reported that >96% of humans use HKQuad in their infancy [14] and that hand-foot crawling is observed rarely [15]. Therefore, we tested HKQuad as a relatively natural locomotor mode for human adults.

In the UpBi task (Figure 2(a), (C)), participants were required to walk bipedally with an upright posture. In this task, the trunk was maintained nearly vertical (high COG) and supported only by two legs (small support base). Compared with the HKQuad task, the posture in this task was considered more unstable because the position of the COG was higher, and the area of the support base was smaller.

In addition to the HKQuad and UpBi tasks, we tested the UpQuad task as artificial quadrupedalism, where participants were required to walk on their two legs and bilateral Lofstrand crutches (Figure 2(a), (B)). In this task, the trunk was maintained nearly vertical (high COG), but it was supported by both the legs and a pair of crutches (large support base) (Figure 2(b)). The postural instability of this task was considered to be intermediate between that of the other two tasks. Compared with the HKQuad task, the area of the support base in the UpQuad task was similar, but the position of the COG was higher, thereby resulting in a more unstable posture, whereas, compared with the UpBi task, the position of the COG was similar, but the area of the support base was larger, thereby resulting in a more stable posture.

Therefore, we were able to rank the tasks in a reasonable manner based on their postural instability, with the UpBi task first, the UpQuad task second, and the HKQuad task last. In addition, as shown in Figure 2(b), we could distinguish the three tasks based on the two aspects of locomotor control: the orientation of the trunk and the number of stepping limbs that encompassed the support base. Therefore, the results could be categorized as one of two cases, as follows. First, if the SMA contributes to the control of upright posture, oxy-Hb levels may be correlated to postural instability and the differences in hemodynamic responses may be altered between tasks performed in an upright posture and those in a horizontal posture (Figure 2(b), left). Second, if the SMA is involved in stepping movements, the results may be altered between tasks performed with four stepping limbs and those with two stepping limbs (Figure 2(b), right).

For safety reasons, the treadmill speed in the HKQuad and UpQuad tasks was set at the slowest (0.8 km/h) for all participants. In the UpBi task, the heart rate was carefully monitored during practice sessions, and the treadmill speed was set for each participant to counterbalance their heart rate during the performance of the HKQuad and UpBi tasks. (We monitored heart rates during the practice sessions and set the speed of UpBi so that the heat rate during HKQuad and the heat rate during the UpBi task would be almost the same.) The actual speed of the 10 participants in the UpBi task ranged from 1.4 to 3.7 km/h (mean 2.3 km/h).

**Figure 3:**
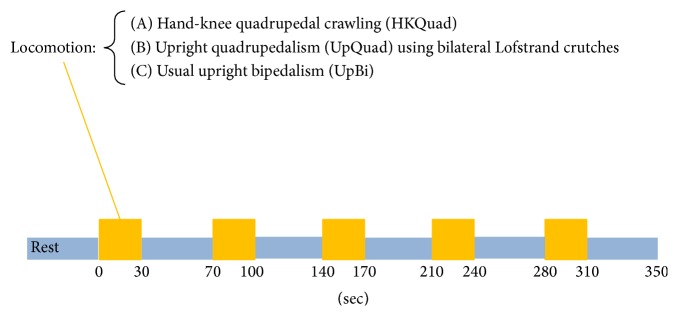
Temporal sequence of the task design. Each task comprised 30 s of locomotion and 40 s of rest, where each pair was repeated five times in total.

Figure 3 illustrates the temporal sequence of the task design. Each task comprised five repetitions of locomotion for 30 s each followed by 40 s of rest as a block. During the rest periods between HKQuad repetitions, participants were instructed to remain still in the hand-knee position. During the rest periods between UpQuad repetitions, they were instructed to stand still while supporting themselves with crutches and two legs. During the rest periods between UpBi repetitions, participants were instructed to stand still while remaining upright.

The order of the three locomotor tasks was counterbalanced among participants. To equalize the head tilt across the three tasks, a mark was placed in front of participants, and they were instructed to look at it during measurements. Body movement artifacts were detected as rapid oxy-Hb change (0.2 mmol/l × mm during 2 s). When body movement artifacts were observed during measurements, these data were excluded from the analyses. We continued the experiments with each participant until we obtained at least three fair repetitions out of five repetitions of each task.

### 2.4. Data Processing

We used oxy-Hb concentrations rather than deoxy-Hb concentrations because the former are reportedly related to brain activity [16–18]. First, the data were automatically processed using the “integral mode,” a command defined by the fNIRS system. This command corrects for baseline drift and averages across 3 to 5 repetitions within each task. A first-degree baseline fit was estimated; the fit was computed between the mean of 5 s period immediately before locomotion and the mean of 30−35 s of the rest period. Thereafter, average of the repetitions was computed. This command is usually recommended for data processing [11, 19, 20]. This process was performed for each channel and for each task in each participant.

Second, we determined the SMA waveform by calculating the spatial average across channels 16, 19, 20, and 23 (i.e., the region of interest covering the SMA). This process was performed for each task in each participant.

**Figure 4:**
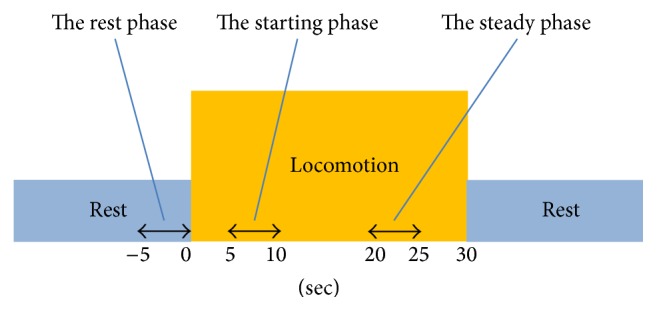
Three distinct phases used in the statistical analyses.

Finally, we calculated the mean oxy-Hb values for three distinct phases during the task: rest, starting, and steady phases (Figure 4). The fNIRS system measured the oxy-Hb value every 0.1 s, and we calculated the temporal mean of a 5 s period for each of the phases. The rest phase oxy-Hb value was calculated as the mean of the 5 s period immediately before locomotion. The starting phase oxy-Hb value was calculated as the mean of the first 5–10 s of the locomotion period. The steady phase oxy-Hb value was calculated as the mean of 20–25 s of the locomotion period. These time periods were selected to consider a delay of several seconds between neural activity and hemodynamic response [21, 22]. It is generally accepted that oxy-Hb values in the starting phase reflect the cortical activity due to adjustment to the initial movement of the treadmill belt, whereas those in the steady phase reflect the cortical activity related to steady locomotion itself [22].

### 2.5. Statistical Analyses

The fNIRS system measures the relative oxy-Hb value compared with the baseline. Therefore, to detect significant hemodynamic changes between rest and locomotion, we performed paired *t*-tests between the rest and the other two phases for all participants according to each locomotor task using SPSS (ver. 22, IBM Corp., NY, USA). Statistically significant differences were accepted at *p* < 0.05.

The postures during the rest periods differed in the three tasks, as mentioned above (Section 2.3); thus we did not compare the oxy-Hb values across the three tasks.

## 3. Results

**Figure 5:**
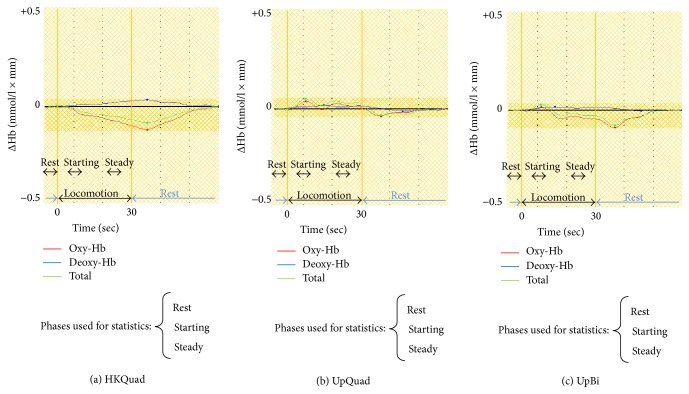
Grand mean oxy-Hb concentration waveforms across all participants. (a) Hand-knee quadrupedal crawling (HKQuad). (b) Upright quadrupedalism (UpQuad) using bilateral Lofstrand crutches. (c) Usual upright bipedalism (UpBi). Waveforms in red, blue, and green represent the concentrations of oxy-Hb, deoxy-Hb, and total Hb, respectively.

Figure 5 shows the grand mean waveforms for the oxy-Hb levels in the SMA across all participants for the HKQuad (a), UpQuad (b), and UpBi (c) tasks. Oxy-Hb values in the rest, starting, and steady phases for each task are shown in Table 1.

In the HKQuad task, the oxy-Hb level progressively decreased from the starting phase until the early rest phase (Figure 5(a)). The waveform of the oxy-Hb level was characterized by a downhill pattern. Compared with the rest phase, the oxy-Hb value in the starting phase was not significantly different, but the value in the steady phase was significantly lower (*p* < 0.05, Table 1).

During the UpQuad task, the oxy-Hb level increased from the beginning of the trial and peaked around the starting phase (Figure 5(b)). Subsequently, the level was maintained near the baseline, followed by a trough in the early rest phase. The waveform of the oxy-Hb level was characterized by a peak-plateau-trough pattern. The oxy-Hb value in the starting phase was significantly higher than that in the rest phase (*p* < 0.05). No significant difference was found between the steady and rest phases.

Finally, in the UpBi task, the oxy-Hb level slightly increased from the beginning of the trial but subsequently decreased and remained below the baseline before a trough in the early rest phase (Figure 5(c)). The waveform pattern of hemodynamic responses observed during the UpBi task was quite similar to that during the UpQuad task (the peak-plateau-trough pattern, Figure 5(b)). However, oxy-Hb values in the starting phase or the steady phase did not differ significantly from those in the rest phase.

In summary, oxy-Hb levels in the SMA significantly decreased during the HKQuad task, whereas they increased during the UpQuad task, with no significant changes in the UpBi task. However, the waveform pattern in the HKQuad task differed from those in the UpQuad and UpBi tasks.

## 4. Discussion

This study investigated hemodynamic responses in the SMA during quadrupedal and bipedal gaits using fNIRS in human adults. Each subject performed three locomotor tasks where the biomechanical instability of truncal posture differed. The present study provides two essential findings. First, the oxy-Hb level significantly decreased during the HKQuad task. Second, statistical analyses showed that the order of oxy-Hb values in the locomotor tasks (UpQuad > UpBi > HKQuad) did not correlate with the order of the postural instability (UpBi > UpQuad > HKQuad). However, qualitative inspection of oxy-Hb time course responses showed that, regardless of quantitative differences, oxy-Hb response patterns differed between the tasks with an upright posture and the task with a hand-knee crawling posture. These results suggest that the task-dependent patterns of hemodynamic waveforms could at least partially reflect the functional significance of the SMA for the control of truncal posture accompanying locomotor movements in humans.

### 4.1. Changes in Oxy-Hb Level during the HKQuad Task

To the best of our knowledge, this is the first study to describe hemodynamic responses in the SMA during quadrupedal gait in humans. During the HKQuad task, oxy-Hb value in the steady phase dramatically decreased beyond the level of significance (Figure 5(a) and Table 1). A decrease in oxy-Hb level is often interpreted as indicating deactivation of a brain region [23–26]. In mammals, the central mechanisms for controlling quadrupedal locomotion largely depend on subcortical structures, and decorticated animals can still walk on a smooth floor without any support [27]. In addition, compared with the quiet stance during rest, crawling involves a hand-knee posture and stepping movements of the arms and legs. Overall, our observation of a decrease in oxy-Hb level suggests that the blood flow over the SMA with a quiet stance could be redistributed to subcortical structures and/or to other cortical areas, such as the primary sensorimotor cortex, during the quadrupedal gait. The current study considered only the SMA because of the limited number of NIRS probes, but we plan to obtain measurements from the primary sensorimotor cortex as well as the SMA in future studies.

### 4.2. Changes in Oxy-Hb Level during the UpBi Task

Previous studies using fNIRS have shown that oxy-Hb level in the SMA significantly increased during bipedal walking [2, 22]. However, in our study, the UpBi task failed to evoke any significant hemodynamic responses in the SMA. At least two possibilities may explain this discrepancy. The first possibility is differences in stepping parameters employed when walking on the treadmill. In our UpBi task, the speed was specifically set for each participant to counterbalance the heart rate between the UpBi and HKQuad tasks, where it ranged from 1.4 to 3.7 km/h. Participants were instructed to walk as comfortably as possible. By contrast, in the study by Miyai et al. [2], the cadence was set at 100 steps/min at 1.0 km/h, which should be considerably higher than that in our study. Mihara et al. [22] also set the speed higher (3.0–5.0 km/h) than that used in the UpBi task in our study. The second possibility is acclimatization to walking on the treadmill. Walking is a rhythmic and stereotyped behavior, which is automatically controlled to a considerable degree at relatively low levels of the nervous system without intervention from higher centers, such as the cerebral cortex [27]. In this study, sufficient practice sessions were conducted prior to measurements to counterbalance the heart rate. Participants may have become acclimatized to walking on the treadmill after these practice sessions, thereby reducing hemodynamic responses over the SMA during the task performance.

Regardless of the statistical significance, the waveform of oxy-Hb responses exhibited a peak-plateau-trough pattern (Figure 5(c)). This response pattern is literally observed not only in the SMA [22] but also in the prefrontal cortex [28, 29] and the medial sensorimotor cortex [28] during bipedal walking by the healthy subjects. This suggests that the peak-plateau-trough pattern might be common features across several cortical regions and that, more importantly, the response pattern observed in the SMA during the UpBi task in this study could not be a simple “noise” but be meaningful. In addition, the increased oxy-Hb level in the starting phase is generally considered to reflect cortical activity due to adjustments of the posture and movements with increasing treadmill speed [22], which also applied to our UpBi task. Thus, it is quite plausible that oxy-Hb concentration responded to the UpBi task but that it remained below the level of significance. This hypothesis is supported by our data obtained from the UpQuad task (see below).

### 4.3. Changes in Oxy-Hb Level during the UpQuad Task

The UpQuad task was the only task where oxy-Hb concentration significantly increased. It is somewhat surprising because walking with a pair of crutches is considered to be more stable than normal walking from a mechanical viewpoint; therefore, we expected that hemodynamic response in the UpQuad task would be lower than that in the UpBi task. Thus, explanations other than postural instability are required. One possible explanation is that because the crutch is an artificial tool, arm movements using the crutch are no longer simple steps; thus, this may be a tool-use behavior involving bimanual coordination, which was reportedly impaired in monkeys with SMA lesions [30].

Our preferred interpretation of the response pattern during the UpQuad task is that the UpQuad task may be considered as a dual task gait, that is, a combination of usual walking (UpBi task) and the putative, bimanual crutch task. Using fNIRS, Mirelman et al. [29] showed that oxy-Hb levels in the prefrontal cortex significantly increased during usual walking while performing a cognitive task, whereas each task alone failed to evoke significant responses. It is not surprising that such facilitatory responses could occur in the SMA. Note again that the hemodynamic response pattern (the peak-plateau-trough pattern) in the UpQuad task (Figure 5(b)) was quite similar to that in the UpBi task (Figure 5(c)). Consequently, it seems as if the waveform in the UpQuad task would be the one, to which the waveform in the UpBi task was shifted upward by the facilitatory effect provided by the putative, bimanual crutch task.

### 4.4. Functional Significance of the SMA in the Control of Bipedal Gait in Humans

Based on statistical comparisons of the three locomotor tasks, we found no correlations between the quantitative changes in oxy-Hb levels in the SMA and postural instability. However, the qualitative patterns of the hemodynamic waveforms were different for the tasks with peak-plateau-trough responses (UpBi and UpQuad tasks) compared with the task with a downhill response (HKQuad task). Mihara et al. [22] also found the peak-plateau-trough pattern during normal walking in healthy subjects. The consistency of the hemodynamic response pattern observed in the SMA among upright locomotor tasks suggests that the SMA may contribute to upright posture control during bipedal gait in humans. Our interpretations are consistent with clinical observations [5, 6], and they support earlier hypotheses regarding the functional role of the SMA [7, 8].

The limitation of this study is that we could not determine the specific role of the SMA in postural control, such as equilibrium and/or weight bearing. To further understand the cortical mechanisms related to upright posture control during locomotion, we will use fNIRS in future research to measure the SMA activity during walking with the body fully stabilized by a body harness (stepping in the air), walking while being burdened on a tilting ground, and walking in zero-gravity conditions in space.

## Figures and Tables

**Table 1:** Oxy-Hb of SMA for each task. Mean and SD of 10 participants (unit: mmol/l × mm).

Task	*n*	Rest phase		Starting phase			Steady phase		
		Mean	SD	Mean	SD	*p* value	Mean	SD	*p* value
Hand-knee crawling (HKQuad)	10	−0.003	0.002	−0.022	0.041	0.1549	−0.069	0.074	0.0178^*∗*^
Lofstrand crutch gait (UpQuad)	10	−0.002	0.003	0.035	0.045	0.0254^*∗*^	0.022	0.082	0.3653
Bipedal walking (UpBi)	10	−0.002	0.003	0.013	0.039	0.2656	−0.033	0.052	0.0968

^*∗*^ *p* < 0.05; *p* values are for paired *t*-test between the rest and starting phases and between the rest and steady phases.

Testing was done within each task.

Oxy-Hb, oxygenated hemoglobin.

SMA, supplementary motor area.

SD, standard deviation.

## Abbreviations

HKQuad: Hand-knee quadrupedal crawling
UpQuad: Upright quadrupedalism using bilateral Lofstrand crutches
UpBi: Upright bipedalism.
